# Successful megaprosthesis in a nearly amputated lower extremity after crush injury: A case report and literature review

**DOI:** 10.1016/j.tcr.2023.100942

**Published:** 2023-10-09

**Authors:** Romy Deviandri, Dhandia Rifardi, Kevin Pratama, Dedi Rahmad Harahap, Gibran Tristan Alpharian

**Affiliations:** a Department of Orthopedics, University of Groningen, University Medical Center Groningen, Groningen, the Netherlands; b Department of Physiology, Faculty of Medicine, Universitas Riau, Pekanbaru, Indonesia; c Department of Surgery, Division of Orthopedics, Faculty of Medicine, Universitas Riau, Arifin Achmad Hospital, Pekanbaru, Indonesia; d Department of Orthopedics, Faculty of Medicine, Universitas Padjadjaran, Hasan Sadikin Hospital, Bandung, Indonesia

**Keywords:** Crush injury, MESS score, Limb salvage, Megaprosthesis

## Abstract

Crush injury is one of the most challenging decisions for a surgeon to decide whether to proceed with an amputation or salvage a limb.

We presented a 24-year-old man who complained of having suffered a crushed thigh 12 h before admission to the hospital. The patient was driving a truck and hit the iron bridge barrier, which penetrated his left thigh. The patient's left foot was cold, pallid, and pulseless, with a MESS score of 11. The femur x-ray showed a displaced fracture of the left femur associated with a 15 cm bone defect. The patient was diagnosed with a crush injury on the left femur with vascular compromise.

We performed a proximal femoral megaprosthesis for a crush injury on the lower extremity, After the sixth year's follow-up, it shows a good outcome and increased quality of life for this patient. In addition, there was an improvement in the Harris Hip Score and EQ5D score.

Megaprosthesis used to treat a crush injury revealed good functional outcomes despite the MESS score of 11. A multi-professional approach to the patient is essential for decision-making regarding limb salvage rather than the use of a score.

## Introduction

**Fig. 1:**
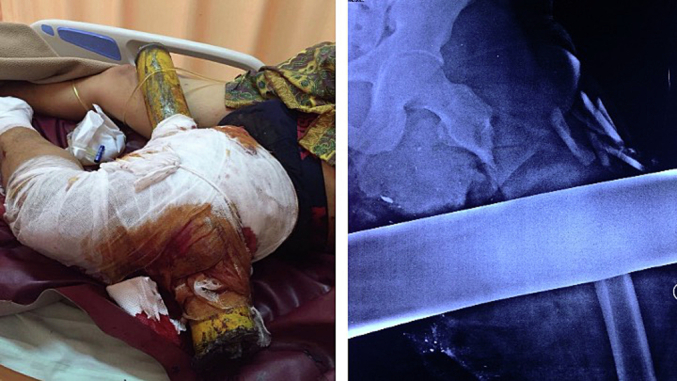
Clinical appearance of the left thigh patient and the left femur x-ray showed a displaced fracture of the left femur associated with a 15 cm bone defect.

Crush injuries are direct trauma and their consequences are caused by compression forces acting on a body part. The severity of the damage caused by crush injuries is influenced by the magnitude and duration of those forces, and severe crush injuries to the extremities can jeopardize the vitality of the limb [1,2]. When faced with these severe limb-threatening injuries, at times the surgeon and patient may have to decide between attempting to salvage or amputate. One of the prognostic tools that have classically been used to aid these decisions is the Mangled Extremity Severity Score (MESS), where scores higher than 7 (out of 11) tend to favour amputation. However, the MESS score is not a clear-cut rule as ultimately it falls on the clinical judgement of the surgeon and shared decision-making with the patient [3]. We report a case in line with the SCARE 2020 guidelines of a limb that was salvaged using an endoprosthesis despite a high MESS score [4] (Fig. 1).

## Case of presentation

**Fig. 2:**
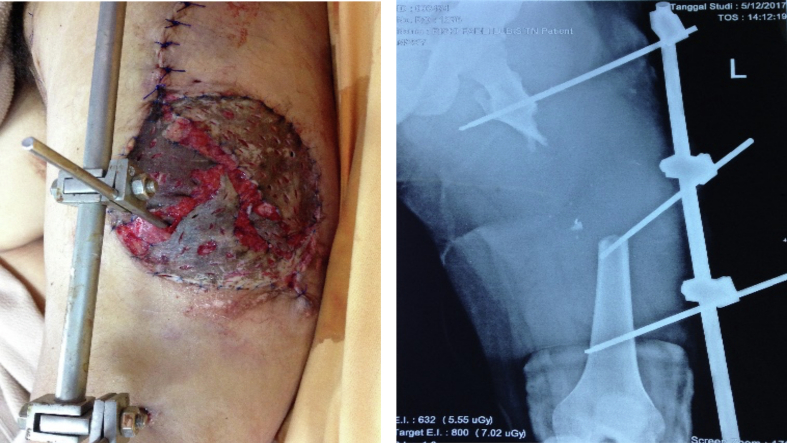
A 9 x 7 cm soft tissue defect located on the left thigh patient and the left femur x-ray showed a displaced fracture of the left femur that had been immobilized by external fixation.

A 24-year-old man presented to the emergency department after sustaining a motor vehicle accident. The truck he was driving collided with the metal railing of a bridge, and as a result of the accident, a cylinder of metal that was part of the railing penetrated his left thigh. On presentation the patient was aware and hemodynamically stable, however, his left lower extremity was cold, pallid, and pulseless below the popliteal fossa. Emergency radiographs taken revealed a severely comminuted subtrochanteric femur fracture with evidence of critical-sized bone loss. Initial examination revealed a MESS score of 11, which entailed amputation, however after deliberation between the surgical team and the patient and family it was decided that an attempt to salvage the limb would be made (Fig. 2).

**Fig. 3:**
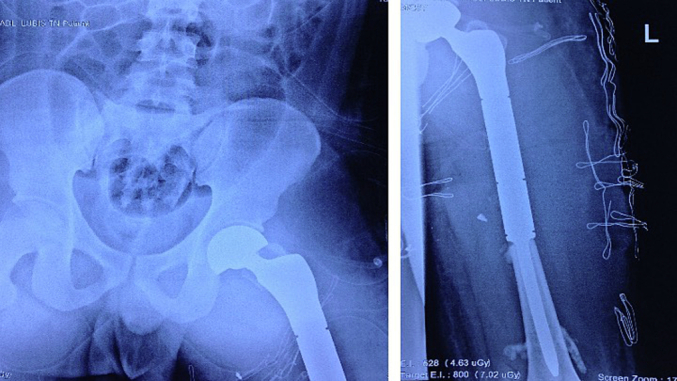
The pelvic and the left femur after installing the proximal femur megaprosthesis.

The initial procedure consisted of evacuation of the metal cylinder and radical debridement. It was found that after careful removal of the offending foreign body and straightening of the limb, the distal pulsation started to return. Intraoperative exploration found defects in the quadriceps muscle group and multiple devitalized fracture segments which were debrided thoroughly, and then the femur was stabilized with an external fixator. A repeat debridement was performed within the next few days, cultures were taken, and the soft tissue defect closed with split-thickness skin grafting (Fig. 3).

After the debridement procedures, the patient was found to have a critically sized bone defect of 15 cm, and thus further discussions were held with the patient and his family. Ultimately we elected to perform an endoprosthesis replacement of the femur after soft tissue healing and laboratory markers, including ESR and CRP returned to normal.

The patient underwent a proximal femur replacement using endoprosthesis which was performed in the lateral decubitus position through a posterior approach extended to the standard lateral approach of the femur. The remaining greater trochanter and gluteus medius were tied on the megaprosthesis through appropriate holes. A few days after the endoprosthesis procedure, there were signs of surgical site infection with pain and discharge from the surgical wound accompanied with fever. Laboratory markers revealed increased CRP and ESR levels. We decided to do a Debridement and Retention of the prosthesis, and The patient was brought back to the operating theatre. Antibiotic-loaded beads were placed after the debridement and cultures were taken, following that, the patient was treated with empirical antibiotics followed by culture-guided antibiotics. A repeat debridement was performed and the beads were removed at two weeks. The patient was then discharged with continued oral antibiotics and regular follow-up at the clinic.

**Fig. 4:**
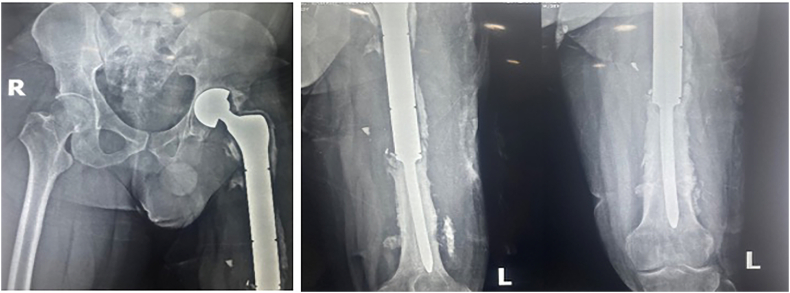
The pelvic X-ray and the left femur X-ray in the sixth year after installing the proximal femur megaprosthesis.

The patient then continued with routine physical therapy. He was able to achieve weight-bearing with crutches 1 month after the surgery and transitioned to independent walking at two years. In the sixth year's follow-up, despite the patient's left knee can only flex to a maximum of 90 degrees, his motor strength is still within normal ranges, allowing him to walk without the use of any assistance. The patient currently has a limp due to an imbalance in leg length, although he did not find this troublesome. A pelvic X-ray examination revealed an acetabular erosion, and the left femur X-ray examination revealed an aseptic loosening process on the bone (Fig. 4). From the sixth year of follow-up, a good outcome is achieved based on the results of the Harris Hip Score and EQ5D questionnaires. The Harris Hip Score increased from 4 to 74.8, and the EQ5D score went from −0,86 to 0.88.

## Discussion

This case report aims to present a limb salvage procedure using endoprosthesis in a patient with a high MESS score of 11. Our case achieved an acceptable outcome for the patient and was able to increase the quality of life at 6 years after surgery. Based on the result of the Harris Hip Score and EQ5d, the patient was able to reach a good outcome.

The MESS score was a predictive tool that is routinely used to help surgeons choose the best course of treatment between salvage and amputation. This score takes into consideration the degree of skeletal and soft tissue injury, limb ischemia, the presence of shock, age, and time of ischemia. The cut-off point is 7, and a score higher than 7 is considered a good candidate for amputation. However, the MESS Score is not a definite rule and must be tailored by the surgeon to the patient at hand. A multi-disciplinary approach to treatment combined with patient-focused decision-making is essential to managing such cases [[3], [4], [5], [6]].

In this case, the patient sustained a 15 cm bone loss due to his injury, and it was one of the factors taken into consideration. The common methods of treating critically sized defects are usually; vascularized bone grafting, the induced membrane Masquelet technique, distraction osteogenesis, or a combination of it [7].

Bone transport using the distraction osteogenesis principle appears to generate reliable outcomes in cases of large critical-sized bone defects. However, the patient may have to endure the long lengthening process which in some patients can be highly painful and uncomfortable. Furthermore, in this case, the proximal short segment is very short and may be difficult to achieve stable proximal fixation, this may jeopardize the bone transfer process [8].

In the masquelet-induced membrane method, the defect is filled first with bone cement and the bone stabilized, and then after a period of time (optimally 6–8 weeks), a second surgery is performed to take the bone cement spacer out and fill the defect using a bone graft. However, for cases where the defect is large (more than 120 mm), the volume of graft needed to be harvested is quite large and this can lead to donor site morbidities, also increased risk of treatment failure. Some experts have also suggested that the Reamer Irrigator Aspirator (RIA) device should be used in large defects to increase the success rate. Furthermore, stability is a significant factor in achieving success using the Masquelet technique and, again, the very short proximal fragment may cause stability issues [7,9].

The vascularized fibular graft can be another option to manage such defects. However, there are problems with the graft size that is harvested, as there is increased morbidity at the donor site. Using a vascularized fibular graft also takes a significant amount of time before the bone remodels into a diameter that matches the original bone, which may lead to a refracture, especially in the lower extremity.

Ultimately, due to socioeconomic conditions and constraints, it was decided that an attempt to salvage using endoprosthesis. The advantage of using endoprosthesis would be the fewer surgeries performed with no additional donor site morbidity. The disadvantage, however, is that endoprosthesis infections continue to be a leading failure mode after limb salvage surgery [10]. After a few of installing the proximal femur endoprosthesis, this patient developed signs of infection accompanied by increased CRP and LED levels. We were able to bring the patient to the theatre again for early debridement and local antibiotic delivery using antibiotic beads, as well as proper culture-guided antibiotic coverage while retaining the stable prosthesis. Early intervention of less than 6 weeks and stable implant were some of the factors that can lead to a successful Debridement and Implant Retention (DAIR) procedure. This has been reported by Gundavda et al. that was successful in controlling endoprosthesis infection by debridement, retention of prosthesis and use of antibiotic-impregnated antibiotic beads for cases of early infections and late acute infections with a stable implant [11].

Long-term use of endoprosthesis is associated with complications and wear processes that reduce their durability. These complications were classified according to the Henderson five-type classification: type 1—soft tissue failures; type 2—aseptic loosening failures; type 3—structural failures; type 4—infection; and type 5—tumour prolapse or progression [9]. On long-term follow-up of this patient, it was found that there were signs of aseptic loosening of the femoral stem. Furthermore, there was significant acetabular erosion that led to a limb length discrepancy and a limping gait. This finding is similar to what Henderson et al. reported that 259 (49 %) of 534 failures were mechanical failures while 102 (19 %) were due to aseptic loosening at the implant-bone interface. The shortest mean time to show up was found for soft tissue failures (16 months), while the longest was found for aseptic loosening (seventy-six months) [11]. Proximal femur megaprosthesis can eventually lead to cartilage damage and acetabular erosion, this was encountered in this patient. If he has extreme pain and radiological cotyloiditis symptoms, a revision surgery to install an acetabular cup can be done to alleviate symptoms [12,13]. However, the patient in this study showed no significant pain until the sixth-year follow-up. We believe that a young patient who dealt prudently with his artificial joints could achieve a good result by keeping his body weight, daily activities, and social environment, which are important factors for achieving good results. This is supported by a study by Liu et al. (2019) that followed a young patient after a partial hip replacement who showed a good outcome after 43 years without significant signs of loosening. In that study, despite the fact that the patient's affected lower leg shortened by 5 cm relative to the opposite side, resulting in a slight limp, and acetabulum wear was noticed, the patient was able to undertake activities of daily living, including walking long distances without pain, and have a good quality of life [14].

## Conclusion

Endoprosthesis can be considered as an option for post-traumatic limb salvage with critical bone defects. It avoids some of the drawbacks that are associated with other options of reconstruction and may result in good functional outcomes despite the high initial MESS score.

## Sources of funding

This case report received no specific grant from public, commercial, or not-for-profit funding agencies.

## Ethical approval

My institution has exempted ethical approval from reporting this case.

## Consent

Written informed consent was obtained from the patient to publish this case report and accompanying images. A copy of the written consent is available for review by the Editor-in-Chief of this journal on request.

## CRediT authorship contribution statement

All Authors have contributed to the case report.

## Declaration of competing interest

The authors have no conflicts of interest to declare.
